# Primary Care Screening and Outcomes for People With Human Immunodeficiency Virus With Poor Care Engagement: The MOD Low-Barrier Clinic

**DOI:** 10.1093/ofid/ofag644

**Published:** 2026-10-05

**Authors:** Raaka G Kumbhakar, Julia C Dombrowski, Katherine Holzhauer, Hayley Yu, Nanki Kaur, Shireesha Dhanireddy, Eve Lake, Audrey Li, Thomas S Fitzpatrick, Maria A Corcorran, Matthew Golden, Jimmy Ma, Jehan Z Budak

**Affiliations:** Division of Allergy and Infectious Diseases, Department of Medicine, University of Washington, Seattle, Washington, USA; Division of Allergy and Infectious Diseases, Department of Medicine, University of Washington, Seattle, Washington, USA; Department of Epidemiology, University of Washington, Seattle, Washington, USA; Public Health–Seattle & King County, Seattle, Washington, USA; Division of Allergy and Infectious Diseases, Department of Medicine, University of Washington, Seattle, Washington, USA; Harborview Medical Center, Seattle, Washington, USA; University of Washington School of Medicine, University of Washington, Seattle, Washington, USA; Division of Allergy and Infectious Diseases, Department of Medicine, University of Washington, Seattle, Washington, USA; Division of General Internal Medicine, Department of Medicine, University of Washington, Seattle, Washington, USA; Division of Allergy and Infectious Diseases, Department of Medicine, University of Washington, Seattle, Washington, USA; Division of Allergy and Infectious Diseases, Department of Medicine, University of Washington, Seattle, Washington, USA; Division of Allergy and Infectious Diseases, Department of Medicine, University of Washington, Seattle, Washington, USA; Division of Allergy and Infectious Diseases, Department of Medicine, University of Washington, Seattle, Washington, USA; Department of Epidemiology, University of Washington, Seattle, Washington, USA; Public Health–Seattle & King County, Seattle, Washington, USA; Division of Allergy and Infectious Diseases, Department of Medicine, University of Washington, Seattle, Washington, USA; Division of Allergy and Infectious Diseases, Department of Medicine, University of Washington, Seattle, Washington, USA

**Keywords:** engagement and retention in care, HIV primary care, low barrier care

## Abstract

This retrospective cohort study evaluated the impact of a low-barrier, walk-in human immunodeficiency virus (HIV) clinic (Moderate Needs [MOD]), on primary care screening and outcomes. Among 462 patients (2018–2024), chronic disease screening, comorbidity care, and viral suppression improved after enrollment in MOD. Low-barrier care for people with HIV can improve both HIV-related and primary care outcomes.

With modern antiretroviral therapy (ART) and increased access to treatment, people with human immunodeficiency virus (HIV-1) in the United States have a life expectancy approaching that of those without HIV [1]. Despite these advances, people with HIV (PWH) remain at higher risk of comorbid conditions due to immune dysfunction, medication effects, and higher rates of coinfections and malignancy [2, 3].

Improving HIV outcomes in the United States requires not only engagement in care and ART access, but also management of comorbidities. As a result, the Health Resources and Services Administration (HRSA) has developed quality measures addressing comorbidity and preventive care [4, 5]. HIV Medicine Association (HIVMA) guidance, updated in 2024, further emphasizes screening, prevention, and management of these conditions [6]. While various published models of low-barrier HIV care have addressed improvements in viral suppression, engagement in care, and comorbid substance use and mental health–related outcomes, none have comprehensively assessed primary care comorbidity care [7, 8].

We previously described the experience of the Moderate Needs (MOD) Clinic, a low-barrier, walk-in clinic for PWH in Seattle, housed within the Ryan White–funded Madison Clinic at Harborview Medical Center at the University of Washington (UW) [9]. The MOD Clinic is part of a broader system of differentiated HIV care that includes several low-barrier clinics and outreach services designed to improve HIV care engagement [8]. MOD was designed to meet the needs of individuals incompletely engaged in conventional HIV care, regardless of viral suppression status, and to address management of HIV-related medical comorbidities [10]. Along the spectrum of differentiated HIV service delivery in King County, the MOD Clinic delivers walk-in primary care from a group of 5 physicians with outreach support from a dedicated program coordinator and the same case management support as in Madison, but with a lower intensity of intervention than the Max Clinic, which additionally provides financial incentives, increased outreach, and case management support [9]. We previously showed enrollment in MOD improved care engagement and sustained viral suppression [9]. However, in our previous analysis, we evaluated only viral suppression and engagement in care; in this evaluation, we assess the extent to which MOD enrollment affected HIV-related primary care process measures and outcomes, and reexamine viral suppression and care engagement in a larger sample.

## METHODS

### Study Population

We conducted a retrospective cohort study among patients enrolled in the MOD Clinic with at least 1 visit from January 2018 (when MOD was established) through March 2024 comparing primary care metrics pre- and postenrollment. Patients who enrolled and subsequently left clinic or died during the follow-up period were included. The population was restricted to patients enrolled through the end of March 2024 to allow for ≥12 months of observation time. Aggregate data were also collected for those years from the Madison Clinic, the Ryan White Clinic in which MOD is housed, for comparison.

### Data Collection

All data were obtained from the UW HIV Information System (UWHIS) and the electronic health record (EHR). For all MOD-enrolled patients, we obtained demographic, laboratory, diagnosis, immunization, and medication order data from UWHIS. Manual chart review of all patients was performed by physicians to ascertain cervical cytology and human papillomavirus (HPV) co-testing results and whether the clinician did lifestyle intervention counseling for hypertension, diabetes mellitus (DM), and dyslipidemia. Yearly aggregate data for the Madison Clinic were obtained from both routine Ryan White and hospital-based quality metrics reporting.

To determine which process measures to include in this analysis, we began with HRSA performance measures and HIVMA primary care guidance routine healthcare maintenance considerations [4–6]. We narrowed these to processes achievable in 1 visit, reasoning that a goal of low-barrier care is to provide primary care intervention or evaluation that would otherwise be excluded in other drop-in visit settings (eg, triage, urgent care, or emergency department visits). Additionally, we excluded any processes with significantly changing guidance during the study period (eg, COVID-19 vaccination, pneumococcal vaccination, anal cancer screening) as this would lead to differences depending on enrollment date.

### Measures

#### Enrollment

Enrollment in the MOD Clinic is determined by the program coordinator and clinicians and is based on factors including poor engagement in care based on HIV viremia and/or multiple missed appointed visits, as described in our prior analysis [9]. Referrals are made by the Madison Clinic (traditional care model) or outside clinicians, case managers, and public health. There are some individuals who are seen in MOD as a “bridge” visit (new diagnoses if there are no available new appointments within the week, those released from jail) but who may not be formally enrolled; these patients are not included in this analysis. Enrollment date was defined as the date of the first MOD Clinic physician visit.

#### Outcomes

The primary outcome was achievement of HIV primary care process measures. These measures included screening for dyslipidemia, DM, hepatitis C virus (HCV), hypertension, and cervical cancer; receipt of influenza vaccination; and HIV viral suppression. Of these, several were defined by laboratory or clinic measurement within 12 months of enrollment: screening for dyslipidemia with lipid panels, DM with hemoglobin A1c or plasma glucose, HCV screening with HCV antibody or RNA, hypertension by blood pressure, and HIV viral suppression, defined as at least 1 HIV-1 RNA <200 copies/mL at any point in the year postenrollment. We collected data on cervical cancer screening for cisgender women and transgender men by chart review, defined by up-to-date age-appropriate collection of cervical cytology with or without HPV co-testing as per guidelines [6, 11]. We also obtained aggregate yearly data for each of these measures for the overall Madison Clinic for comparison; these were not available for cervical cancer screening. Visit data for engagement in care, defined as at least 2 visits at least 90 days apart in the 12 months pre– and post–MOD enrollment, were collected.

Secondary outcomes included interventions prescribed or performed for comorbid conditions (new or preexisting) in the year following enrollment at the MOD Clinic, including hypertension, dyslipidemia, DM, and HCV. Diagnoses of hypertension, DM, and dyslipidemia were determined using definitions from the Centers for AIDS Research Network of Integrated Clinical Systems (CNICS) [12], and confirmed by physician chart review. HCV diagnoses were determined by a positive HCV RNA test in the year following enrollment at the MOD Clinic. Pharmacologic intervention for any diagnosis was determined using medication order data. Lifestyle intervention was determined through physician chart review and defined as nonpharmacologic measures as documented in chart notes, including counseling on or achievement of dietary changes, exercise, substance use cessation, etc.

### Statistical Analysis

#### Primary Analysis

For each primary care process measure, we determined the proportion of patients who achieved the measure in the year prior to their enrollment date at the MOD Clinic and the year following enrollment. Day of enrollment was included in the postenrollment period. McNemar χ^2^ test was used to compare the proportion of patients who achieved the measure in the year preenrollment to the proportion who achieved the measure in the year postenrollment; Bonferroni correction was used to adjust *P* values to account for multiple comparisons.

We conducted a sensitivity analysis to assess the impact of 1-time patients in MOD Clinic (ie, with no subsequent follow-up visits in which to address primary care issues) on the primary care process measures. This was done to account for patients with only 1 visit and who had a process measure obtained in that visit but never returned for potential intervention. Patients visiting the clinic only on the day of enrollment were excluded, and results were compared with those of the primary analysis.

#### Secondary Analysis

Among those MOD patients with diagnoses of hypertension, DM, and dyslipidemia, we determined the proportion who received pharmacologic intervention and/or lifestyle intervention; for those diagnosed with HCV, we found the proportion who received treatment.

R version 4.4.2 was used to conduct all statistical analyses. The University of Washington Human Subjects Division approved this study (STUDY00018126).

## RESULTS

A total of 462 patients were enrolled in the MOD Clinic between January 2018 and March 2024. Most patients were male (85%), and non-Hispanic (71%); 61% were white, and 29% were Black. The average age of the patients at enrollment was 42 years (Supplementary Table 1).

For all primary care process measures evaluated, there was a statistically significant higher proportion of patients who achieved the measure in the postenrollment period (Table 1). Compared to the range of process measures performed annually during 2019–2024 among all Madison Clinic patients, proportions achieved among MOD patients such as diabetes screening (49% vs 39%–53%), HCV screening (59% vs 31%–36%), influenza vaccination (47% vs 43%–49%), and blood pressure measurement (97% vs 89%–99%) were similar or better. Viral suppression increased from 58% in the 12 months preenrollment to 84% in the 12 months postenrollment (*P* < .001), the latter of which was comparable to viral suppression rates in the overall clinic during the study period (83%–90%).

**Table 1.** Proportion of Madison and Moderate Needs (MOD) Clinic Patients Who Achieved Screening Measures in the 12 Months Preenrollment and 12 Months Postenrollment (N = 462)

Measure	Range of Madison Clinic Metrics (2019–2024)	12 Months Preenrollment, No. (%)	12 Months Postenrollment, No. (%)	*P* Value^a^
Lipid panel	54%–62%	145 (31%)	218 (47%)	<.0001
Diabetes screening	39%–53%	125 (27%)	228 (49%)	<.0001
HCV screening	31%–36%	188 (41%)	274 (59%)	<.0001
BP measurement	89%–99%	345 (75%)	450 (97%)	<.0001
Cervical cancer screening up to date (n = 58 eligible)	…	20 (34%)	35 (60%)	.005
Influenza vaccination	43%–49%	128 (28%)	216 (47%)	<.0001
Engagement in care^b^	…	127 (27%)	340 (74%)	<.0001
Viral suppression^c^	83%–90%	269 (58%)	385 (83%)	<.0001

Abbreviations: BP, blood pressure; HCV, hepatitis C virus.

^a^ *P* values are adjusted for multiple comparisons with the Bonferroni correction.

^b^Two or more provider visits ≥90 days apart in the time period.

^c^At least 1 HIV-1 RNA <200 copies/mL at any point during the time period.

Of the study population, 314 (68%) had at least 1 visit in the Madison Clinic prior to enrollment; 148 (32%) had no visit in the year prior. In the 12 months postenrollment, 390 (84%) had at least 1 follow-up visit; 72 (16%) had no follow-up. Engagement in care increased from 27% to 74% in the postenrollment period.

In the sensitivity analysis restricting the population to those with >1 visit in MOD, 99 of 462 (21.4%) of patients were excluded for having only an enrollment visit to MOD. There was no significant difference in the proportions of patients achieving each process measure as compared to the primary analysis.

During the study period, 104 (23%) patients had hypertension, 77% of whom received either counseling or pharmacologic intervention; 27 (6%) had DM, 85% of whom had any intervention; 53 (11%) had dyslipidemia, 25% of whom had any intervention; and 47 (10%) had HCV (9 [19%] were prescribed treatment, 7 of whom achieved sustained virological response) (Table 2).

**Table 2.** Proportion of Moderate Needs (MOD) Clinic Patients With Comorbid Conditions and Interventions Prescribed or Performed in the Postenrollment Period (N = 462)

Comorbid Condition	No. (% of All Patients)	Either or Both Interventions	Pharmacologic	Lifestyle Modification Counseling
Hypertension	104 (23%)	80 (77%)	77 (74%)	25 (24%)
Diabetes mellitus	27 (6%)	23 (85%)	19 (70%)	18 (67%)
Dyslipidemia	53 (11%)	13 (25%)	10 (19%)	6 (11%)
Hepatitis C	47 (10%)	9 (19%)	9 (19%)	…

## DISCUSSION

Patients who enrolled in a low-barrier, walk-in primary care HIV clinic, previously shown to improve care engagement, had improved comorbidity care and preventive care. We found statistically significant increases in primary care process measures after enrollment, and high rates of counseling and/or pharmacologic intervention on most diagnosed comorbid conditions after enrollment. While the latter was not the case for dyslipidemia or hepatitis C, importantly, for screening for dyslipidemia, DM, hepatitis C, hypertension, and influenza vaccination uptake, MOD enrollment met or exceeded Madison Clinic (ie, conventional model) metrics. Additionally, these reflected outcomes only in the first 12 months postenrollment, and in a longer follow-up time period those interventions may have been achieved. We found a statistically significant increase in engagement in care, consistent with our prior analysis; a majority had at least 1 MOD follow-up visit in the year following their enrollment visit. While it may seem evident that engagement in care would necessarily lead to improvements in primary care, this population has complex needs in which comorbidity screening and intervention may not be prioritized in a nonstructured setting.

Viral suppression increased in the preenrollment to postenrollment period. Unlike in our previous analysis, the difference was statistically significant [9]. In this analysis, the MOD population was substantially larger (462 vs 164) and fewer patients were virally suppressed at enrollment (58% vs 63%). The significant increase in viral suppression thus likely reflects (1) the larger cohort—when applying the prior pre- and postenrollment viral suppression rates to the sample size in our current analysis, the increase in viral suppression in the prior analysis reached statistical significance; and (2) increase in referrals of individuals more likely to be virally unsuppressed at time of referral but able to achieve suppression once engaging in care. In addition, programming in MOD became more robust with the establishment of a core group of clinicians, the addition of a program coordinator, and multisector coordination enhancing care engagement among referred patients.

A key strength of our study was the inclusion of nonpharmacologic interventions from chart review, capturing a broader scope of care and enriching our primary outcome by demonstrating whether interventions subsequently occurred for the comorbidities for which patients were screened. Data were limited to those abstracted from UWHIS and the EHR; while these are robust, we could not capture vaccinations or screenings obtained outside of the UW system. However, the goal of this study was to evaluate services provided in the MOD Clinic, not in the healthcare system at large. In the secondary analysis, pharmacologic intervention data were limited to the postenrollment period and did not account for continuation or adjustment of existing therapies with exception of HCV, in which pharmacologic treatment is time limited. Additionally, there was no comparator group, limiting the extent to which we can understand MOD's impact on these outcomes. Further, given the retrospective pre-post study design, we cannot say with certainty which outcomes may have changed with or without MOD. Overall Madison metrics, however, provide a benchmark for comparison to usual care over the study period. While we had no pre-post comparison for the secondary outcome of comorbidity intervention, these were individuals not in care prior to MOD and unlikely to have had previous meaningful engagement for those diagnoses.

Nearly 7% (500) of all PWH in King County are enrolled in MOD [13]. Over the course of 8 years, the clinic has proven to be a scalable and sustainable intervention, dependent on Ending the HIV Epidemic funding and a success of that program. Assuming a dedicated funding mechanism, the MOD Clinic model is an exportable HIV primary care intervention.

These findings inform ongoing discussions of the role of the infectious diseases/HIV clinician as a primary care versus specialty provider for PWH and highlight the potential for integrated, low-barrier care approaches to improve health outcomes beyond HIV [14, 15]. While this does not settle the question, we highlight a population with complex needs for whom care may only be feasibly delivered in 1 setting. In this case, individuals with barriers that required the MOD Clinic to improve engagement had a clear benefit with respect to primary care; further fragmenting care could lead to deleterious impacts on both their HIV and comorbidities.

In summary, low-barrier HIV care was associated with improved primary care process measures, comorbidity management, and viral suppression. These findings support integrated, low-barrier models as effective strategies to address both HIV outcomes and broader health needs.

## Supplementary Material

ofag644_Supplementary_Data

## Supplementary Data

Supplementary materials are available at *Open Forum Infectious Diseases* online. Consisting of data provided by the authors to benefit the reader, the posted materials are not copyedited and are the sole responsibility of the authors, so questions or comments should be addressed to the corresponding author.

## References

[1] Marcus JL, Leyden WA, Alexeeff SE, et al Comparison of overall and comorbidity-free life expectancy between insured adults with and without HIV infection, 2000–2016. JAMA Netw Open 2020; 3:e207954. 10.1001/jamanetworkopen.2020.7954 PMC7296391 32539152

[2] Gallant J, Hsue PY, Shreay S, Meyer N. Comorbidities among US patients with prevalent HIV infection—a trend analysis. J Infect Dis 2017; 216:1525–33. 10.1093/infdis/jix518 29253205

[3] Wong C, Gange SJ, Moore RD, et al Multimorbidity among persons living with human immunodeficiency virus in the United States. Clin Infect Dis 2018; 66:1230–8. 10.1093/cid/cix998 PMC5889007 29149237

[4] Horberg MA, Certa JM, Rubenstein KB, et al Beyond the HIV care continuum and viral suppression: broadening the scope of quality metrics for total HIV patient care. AIDS Patient Care STDS 2020; 34:461–9. 10.1089/apc.2020.0145 PMC7698986 33147087

[5] Ryan White HIV/AIDS Program. Performance measure portfolio . **2025**. Available at: https://ryanwhite.hrsa.gov/sites/default/files/ryanwhite/about-program/hab-core-performance-measures.pdf. Accessed 5 October 2026.

[6] Horberg M, Thompson M, Agwu A, et al Primary care guidance for providers who care for persons with human immunodeficiency virus: 2024 update by the HIV Medicine Association of the Infectious Diseases Society of America [published online ahead of print 12 October 2024]. Clin Infect Dis 2024. doi:10.1093/cid/ciae479 39393187

[7] Imbert E, Hickey MD, Clemenzi-Allen A, et al Evaluation of the POP-UP programme: a multicomponent model of care for people living with HIV with homelessness or unstable housing. AIDS 2021; 35:1241–6. 10.1097/QAD.0000000000002843 PMC8186736 34076613

[8] Dombrowski JC, Ramchandani MS, Golden MR. Implementation of low-barrier human immunodeficiency virus care: lessons learned from the Max Clinic in Seattle. Clin Infect Dis 2023; 77:252–7. 10.1093/cid/ciad202 PMC10371304 37021670

[9] Kumbhakar RG, Budak JZ, Tao Y, et al The impact of a walk-in human immunodeficiency virus care model for people who are incompletely engaged in care: the Moderate Needs (MOD) Clinic. Open Forum Infect Dis 2023; 10:ofac670. 10.1093/ofid/ofac670 PMC9825197 36628059

[10] National Institutes of Health. Human papillomavirus disease: adult and adolescent OIs . 2024. Available at: https://clinicalinfo.hiv.gov/en/guidelines/hiv-clinical-guidelines-adult-and-adolescent-opportunistic-infections/human. Accessed 24 April 2026.

[11] Kitahata MM, Rodriguez B, Haubrich R, et al Cohort profile: the Centers for AIDS Research Network of Integrated Clinical Systems. Int J Epidemiol 2008; 37:948–55. 10.1093/ije/dym231 PMC2597168 18263650

[12] Public Health–Seattle & King County. HIV/AIDS data—King County, Washington . 2025. Available at: https://www.kingcounty.gov/en/dept/dph/health-safety/disease-illness/hiv-sti-hcv/data-reports/annual-reports. Accessed 24 April 2026.

[13] Lakshmi S, Beekmann SE, Polgreen PM, Rodriguez A, Alcaide ML. HIV primary care by the infectious disease physician in the United States—extending the continuum of care. AIDS Care 2018; 30:569–77. 10.1080/09540121.2017.1385720 PMC5967237 28990409

[14] El-Sadr WM, Platt J. The promise of integration of HIV into primary care: challenges and opportunities. J Int AIDS Soc 2026; 29:e70093. 10.1002/jia2.70093 PMC12962869 41786660

