# Supplementary material for: Primary Care Screening and Outcomes for People With Human Immunodeficiency Virus With Poor Care Engagement: The MOD Low-Barrier Clinic
Source: Open Forum Infect Dis. 2026 Oct 5;13(10):ofag644. doi: 10.1093/ofid/ofag644 (PMC13646599; doi:10.1093/ofid/ofag644)
Supplement: Supplementary file 1 [file ofag644_supplementary_data.docx]

**Supplementary Table 1.** Characteristics of patients enrolled in the MOD Clinic (N=462), from April 1, 2018 to April 1, 2024

|  | **MOD patients**  **N = 462** |
| --- | --- |
| **Age at enrollment** | 42 (±12) |
| **Gender** | |
| Male | 395 (85%) |
| Female | 61 (13%) |
| Transgender | 6 (1%) |
| **Ethnicity** | |
| Hispanic or Latino/a | 65 (14%) |
| Non-Hispanic or Non-Latino/a | 329 (71%) |
| Unknown or declined | 68 (15%) |
| **Race** | |
| White | 281 (61%) |
| Black of African American | 132 (29%) |
| Asian | 11 (2%) |
| American Indian | 20 (4%) |
| Native Hawaiian or other Pacific Islander | 11 (2%) |
| Multiracial | 3 (1%) |
| Unknown or declined | 4 (1%) |
